# 3D Technology Used for Precision in Orthodontics

**DOI:** 10.7759/cureus.47170

**Published:** 2023-10-17

**Authors:** Samiksha R Thawri, Priyanka Paul, Amit Reche, Harsha P Rathi

**Affiliations:** 1 Department of Public Health Dentistry, Sharad Pawar Dental College and Hospital, Datta Meghe Institute of Higher Education and Research (Deemed to be University), Wardha, IND

**Keywords:** treatment outcomes, virtual planning, three-dimensional, efficiency, orthodontics, effectiveness

## Abstract

One of the most crucial technologies used by orthodontists to assess and document the dimensions of craniofacial features is imaging. Orthodontists frequently employ two-dimensional (2D) imaging methods, although 2D imaging cannot localize or determine the depth of structures. Early in the 1990s, three-dimensional (3D) imaging was invented, and it has since become a crucial part of dentistry, especially in orthodontics. One of the newest and most important breakthroughs in dentistry is 3D technology. Clinicians have been able to significantly improve patient care while also shortening the time spent on treatment planning due to these technologies, which include intra-oral scanning, 3D imaging, computed-axial tomography (CAT) scan, cone-beam computed tomography (CBCT), computer-aided design/computer-aided manufacturing (CAD/CAM), and 3D software. 3D models of maxillary and mandibular arches can take the place of conventional plaster casts and their limits for planning treatments, appliance production, and estimated treatment results as part of this continuous progress. Digital orthodontics procedures have become more popular in the recent past. The development of “personalized” orthodontic appliances makes use of technology. These technologies' overall improvement can increase clinicians' productivity and efficiency by simplifying traditional methods that are seen to be particularly laborious. The objectives of this review are to provide an overall description of the 3D technology nowadays and to assess its orthodontic applications.

## Introduction and background

The use of three-dimensional (3D) technologies is widespread in several dental specialties, including orthodontics. The adoption of these tools has steadily altered how clinicians conduct diagnostic, treatment plans, case monitoring, and outcome evaluation in both orthodontics and maxillofacial surgery. To accurately display tridimensional anatomy, these technologies duplicate anatomical structures [1,2]. For an accurate diagnosis and plan of treatment to be provided, a dental examination and assessment of the facial hard and soft tissues are essential. Two-dimensional (2D) imaging methods are frequently used by orthodontists to record craniofacial anatomy. The identification of retained teeth, facial asymmetries, distortions of cephalometric points away from the mid-sagittal axis, and facial asymmetries are limitations of these techniques. The spread of 3D imaging technology led to the development of methods that enable users to analyze 3D data and as a result, conduct more complicated case studies on a craniofacial level.

Although effective, these methods are not generally utilized. This scenario can be related to a number of things, such as clinicians' lack of understanding of these advances, the learning process necessary for operating the systems, the challenges in the qualitative evaluation related to the development of software, and the radiation dose required to obtain an entire facial image [3-5].

Cone-beam computed tomography (CBCT), stereophotogrammetry, the digitalization of 3D devices used for intraoral scanning, and devices for 3D printing using computer-aided design/computer-aided manufacturing (CAD/CAM) technology are the most widely used 3D techniques in orthodontics. These printers can be used to create transparent aligners, customized orthodontic braces, surgical guides, and indirect bonding trays [6].

Applications for orthodontic CAD/CAM today include lingual appliances, titanium Herbst appliances, clear aligner therapy, and tools for diagnosis and treatment planning [7]. Robotically bent archwire, machine-milled indirect bonding jigs, and customized brackets with patient-specific torque are among the newest CAD/CAM innovations in the field. “Improving predictability, efficiency, and quality of orthodontic treatment” might best be summed up as the ultimate goal of using CAD/CAM technology in the area of orthodontics [8].

The use of 3D technology enables the exact and personalized machining of orthodontic appliances as well as the use of virtual treatment planning software by the practitioner and patient to more clearly define case objectives and see treatment outcomes. Practitioners can contrast various treatment strategies. Improved communication between healthcare professionals and patients leads to more reasonable expectations for treatment outcomes and a higher level of trust between the two parties [9,10]. The rationale of this article is to analyze the potential uses of 3D technology in orthodontic diagnosis, treatment planning, case monitoring, and outcome evaluation.

## Review

Search methodology

**Figure 1:**
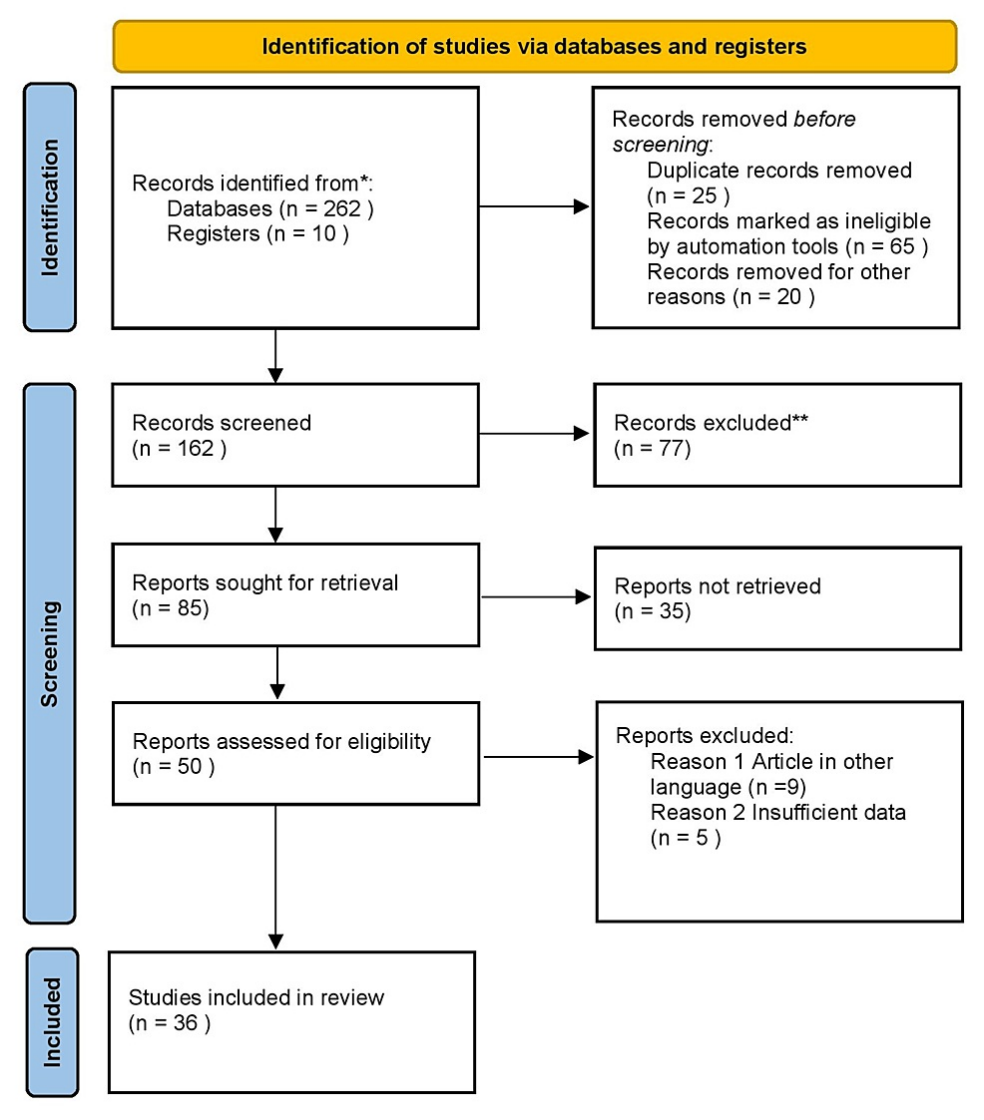
PRISMA flow chart of search strategy

We conducted a review through PubMed and Google Scholar referring last 10-year articles in June 2022 using keywords such as “virtual planning,” “three dimensional,” “efficiency,” “orthodontics”, “effectiveness” and “treatment outcomes.” We additionally searched for key references from bibliographies of the relevant studies. The search was updated in April 2023. The reviewer monitored the retrieved studies against the inclusion and exclusion criteria in the beginning based on the title and abstract and then on full texts. For inclusion, both published and unpublished studies in the English language were considered. We excluded studies published in other languages because of resource limitations and full-text articles were unavailable to the reviewer. Figure 1 shows the PRISMA flow diagram for the selection process of the article.

Diagnosis

3D Cephalometry and CBCT Assessment

Cephalometric evaluation, which assists in determining dental as well as skeletal correlations in the craniofacial complex, is a fundamental tool in the field of orthodontics. In the past times, computed-axial tomography (CAT) scanners were frequently used in dental clinics to diagnose patients. However, concerns about the patient's exposure to high doses of radiation were taught about, leading to the development of the CBCT as a lower radiation option in 1996 [11]. A CBCT examination is used to do 3D cephalometry, which enables a more thorough analysis of the craniofacial structure. The dentist can more quickly identify and measure longitudinal growth, minor occlusal alterations, and craniofacial discrepancies with this method [12,13]. Recent research has also shown that 3D diagnostic imaging not only is precise but is also comparable to classic cephalometric analysis and direct cranium measurements [14].

Method of Airway Measurement Using CBCT

With the use of a CBCT, accurate 3D airway measurements can be obtained. Patients suffering from obstructive sleep apnea syndrome have found this to be a valuable tool for comparing the narrowing of the airways prior to and following various methods of treatment. Various points and planes can be used to define and then measure the pharyngeal region. The superior plane runs from the basion to the posterior nasal spine, the medial plane runs in line with the base of the cranium plane and passes through the medial point in the first vertebra, and the inferior plane runs through the anteroinferior point of the first vertebra to the ment, these are the three planes that Cossellu et al. divided in this region [15]. The retropalatal space, which runs from the posterior nasal spine to the inferior edge of the soft palate, and the retroglossal space, which runs from the inferior edge of the soft palate to the hyoid bone, were combined in an alternative evaluation approach provided by Schendel et al. [16]. Brunetto et al. proposed volume of airway measurements using the combining of two sectors. Anatomical limitations, such as the sphenoidal sinus and cervical vertebrae, and planes that are perpendicular to or parallel to the Frankfort Horizontal Plane are used to establish Volume A, the upper sector. The lower sector, Volume B has the same anterior and posterior boundaries as Volume A, but its upper limit also corresponds with Volume A's lower limit, and its lower limit is traced using a line parallel to the Frankfort Horizontal Plane that passes through the fourth vertebra's anteroposterior point [17]. When the upper jaw expands, the nasal cavity might undergo alterations. Linear measures are collected from the first premolar, first molar, and nasal base width in an effort to evaluate these changes. The first premolar's distance between cuspids or between root apex is calculated. The distance between either the disto-vestibular cuspids or the apexes of the vestibular roots is calculated while evaluating the first molars. Calculating the most extreme left and right areas where the upper sinus and the nasal cavity intersect, can be used to measure the nasal base width [18]. These techniques have been demonstrated to be efficient in the examination of airways and are a crucial tool in the precise evaluation of therapy efficacy.

Assessment of Synchondrosis Using 3D Imaging

**Figure 2:**
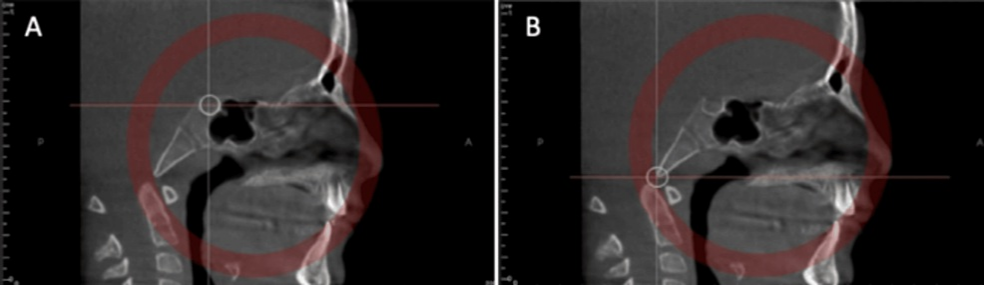
Spheno-occipital synchondrosis ossification assessment: Middle of sella turcica in the sagittal view (A). Anterior border of the foramen magnum in the sagittal view (B). Copyright: © 2022 by the authors. Licensee MDPI, Basel, Switzerland. This article is an open access article distributed under the terms and conditions of the Creative Commons Attribution (CC BY) license (https:// creativecommons.org/licenses/by/ 4.0/).

The sphenoid-occipital synchondrosis is the final cartilage to fuse in the body, joining the sphenoid bones' bodies to the basilar region of the occipital bone. The development of this structure will affect the cranium's anteroposterior dimension, and inappropriate fusion might result in malocclusion. The synchondrosis stage of ossification can be assessed by CBCT imaging. There are various planes involved in the process used to establish the midsagittal plane for this evaluation. The first two planes intersect the foramen magnum at its anterior border with one passing through the axial plane and the other in the sagittal plane whereas the other two planes pass through sella tunica in axial and sagittal planes (Figures 2A, 2B). These can aid in the prediction of potential development patterns and assist in formulating treatment strategies that could reduce any negative outcomes [19].

Treatment Diagnosis Using a 3D Scanner

In 1999, Cadent created the first orthodontic digitation system, and since then, the technology has advanced significantly. With this technique, the dentist can scan the oral cavity or use plaster models or impressions to directly construct a 3D image of the upper and lower arch, alone or in the occlusion. When compared to their conventional counterparts, digital study models have a few advantages, including improved tolerance, increased comfort, reduced allergy risk, as well as simple data storage, recovery, and peer sharing. Orthodontic 3D models and plaster models were used in certain research to evaluate measuring sensitivity and diagnosis accuracy [20]. Digital models are not only extremely accurate and dependable but also simple to replicate, according to the systematic study conducted by Rossini et al. [21].

Orthodontic treatment and monitoring

Indirect Direct Bonding With 3D Imaging 

Techniques for indirect bracket bonding have now been designed to decrease human error caused by changes in the anatomic and biological shape of crowns. Due to the CBCT's 3D view of the individuals' hard and soft tissues, the advancements in imaging techniques have enhanced diagnosis and treatment. When bonding 3D photos of the 3D image to better visualize the crown and roots. The projection is then enhanced with a picture of a “U” molding tray, which is then modified to fit the teeth and brackets. The upper half of the teeth and the brackets will be covered by this molding tray, leaving the other half exposed. Following the removal of the bracket and tooth images, we are then left with the guide for bonding that is prepared for the printing process. Indirect bonding can occur after printing and tray adjustment, reducing human error [22].

Manufacturing of Clear Aligner

Clear aligners are built on technologies that depend extensively on 3D technology, particularly CBCT and intraoral scanners. The orthodontist can utilize the intraoral scanner to produce 3D images of the patient's oral cavity and set up the correct orthodontic motions using a particular automated soft tissue adjustment program. In a single clear aligner case, many trays are required to provide the desired outcome. The aligner sequence causes progressive orthodontic movements. There are distinct models and, consequently, a separate tray for each movement. The orthodontist must specify the desired results on the program following the diagnosis before beginning treatment. The orthodontist must split the intended dental motions at this stage to determine the total number of aligners required to achieve the desired outcomes. Each model in 3D is transformed into a file known as a Standard Triangle Language (STL) once all the projections have been made so they can be 3D printed. The aligners will subsequently be produced using thermoformed plastic using these models.

Surgical Guide for Mini Screw Placement

Miniscrews create skeletal anchoring, simplifying several treatment strategies. There has been a surge in the usage of these devices in orthodontics as they are simple to insert and remove and do not require the patient's cooperation. However, the stability is mostly dependent on bone density [23]. Before inserting any mini-screws, orthodontists must carefully evaluate each case because bone thickness differs from person to person. By removing problems with 2D imaging, the introduction of 3D imaging tests has enhanced diagnosis and treatment strategies [24]. To determine the ideal location for mini-screws, digital models superimposed on CBCT data can be used. A surgical guide can be produced using a 3D printer after the appropriate placement site has been chosen. These guides will enable precise and regulated insertion while also reducing the hazards that are frequently connected to this process [25].

3D Printed Surgical Guides for Corticotomy Technique

 A corticotomy can be described as a surgical procedure that intentionally inflicts mechanical damage on the cortical bone to lower its resistance and significantly shorten the course of treatment. This approach is believed to be the only relatively low-risk and effective treatment that accelerates the movement of teeth. In this method, which is also known as corticotomy-assisted orthodontic therapy (CAOT), tiny incisions are made along the alveolar bone wherein movement is intended to occur.

Tooth Movement Assessment in the 3D Plane

A new methodology proposed that the orthodontist would have been able to evaluate the root location at any moment without subjecting the patient to additional radiation by integrating CBCT with intraoral imaging [26]. The orthodontist finds it convenient to employ digital orthodontic models since data can be transferred, recovered, and stored with reasonable ease. Models are easy to use for measuring the mesiodistal crown dimension, crowding of teeth in the dental arch, and arch length, facilitating a quicker diagnosis process, particularly in cases involving extractions. Impressions are made both before and after treatment to accurately assess dental mobility. The models that are produced are then digitalized with a 3D scanner and exported as the STL file.

Orthognathic Surgery 3D Planning Surgical Splint

**Figure 3:**
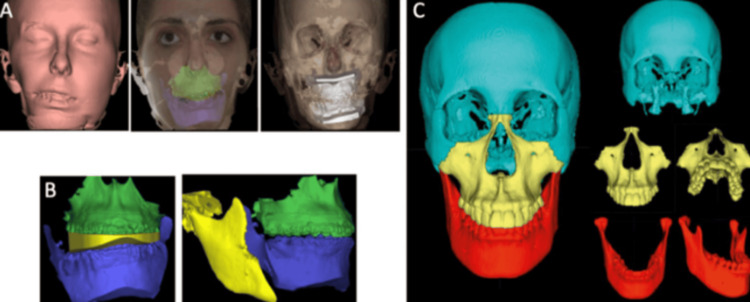
3D orthognathic surgery planning: Preoperative virtual simulation (A). Predicted results on hard tissues after repositioning the mobilized bone structures: intermediate virtual splint and final occlusion (B). Postoperative virtual simulation of the predicted results on hard tissues after repositioning the mobilized bone structures (C). Copyright: © 2022 by the authors. Licensee MDPI, Basel, Switzerland. This article is an open access article distributed under the terms and conditions of the Creative Commons Attribution (CC BY) license (https:// creativecommons.org/licenses/by/ 4.0/).

Patient photographs and cephalograms are used as a reference for 2D computer-assisted surgical systems used for planning conventional orthognathic operations [27,28]. These technologies are combined with the use of a facial arch to properly register the patient's bite using a semi-adjustable articulator. To create surgical splints, this approach enables the modeling of surgical motions utilizing cast models of the patients [29]. Despite being highly developed and widespread, this technology has certain drawbacks since employing a traditional articulator and planning 3D treatments using 2D imagery might lead to errors. Some of the problems with 2D modeling previously described have been addressed by the development of 3D simulation systems using CBCT. These procedures necessitate a CBCT, which must be performed with the patient's head and muscles in a natural position with an expression of relaxation while biting in centric relation. By mapping 2D images, utilizing 3D photographs, or combining 3D surface scans using CBCT reconstruction data, the texture of the skin and structures may be enhanced and refined in order to precisely overlap with the CBCT reconstructions. This refinement may be done for intraoral structures and dental artifacts by digitizing plaster casts, directly scanning intraoral objects in three dimensions, and scanning dental impressions using surface-based laser scanning. To confirm that there are no problems with the rendering procedure, scanning must be performed within minutes following the CBCT. Precision is required while obtaining the bite registration, and numerous bite recordings should be taken to avoid errors when there are functional deviations or duplicate bites that cause several disruptions [30]. By moving each segment in reference to all three spatial planes (x, y, and z) using this method, changes are accomplished by rotating the segments along the axis that represent “roll, pitch, and yaw.” Additionally, essential nearby structures that could obstruct the osteotomy procedure can be found, including the sinus of the maxilla and inferior alveolar nerve. The surgical splints can be developed using computer-aided design (CAD) methods following 3D planning for surgery. The 3D program may be used to design guide models to anticipate surgical incisions as well as the placement of screws and surgical plaques (Figures 3A-3C) [19].

The approach seems to possess a couple of advantages over the traditional approach, including the ability to recognize certain distortions or asymmetries that might have otherwise remained undetected, the capacity to simulate multiple procedures during surgery, the capacity to identify possible problems, and the capability to correct the centric relation position on TMJ level [31]. This technique also has the benefit of making information sharing with other clinicians easy. Although a few of these 3D technologies and methods are discussed in the most recent research, neither among them has been referred to as being an accepted standard for picture superimposition [32].

Customized Wires

Historically, brackets with a specialized tip and torque have received major attention on the topic of personalized equipment. About CAD/CAM orthodontics, OraMetrix® has adopted a distinctive strategy that focuses on wires. CAD/CAM technology in orthodontics, OraMetrix®'s Sure Smile TM offers computerized programming that the orthodontist can use for diagnosis and treatment planning, but what sets Sure Smile apart from other bespoke appliances is the production of robotically bent arch wires [33]. The Sure Smile system can be used with traditional brackets because the technique relies on the production of the accuracy of archwires of different sizes, thus no extra considerations need to be made when the appliances are delivered. Through the use of intraoral scanners or CBCT, the patient's dentition is first scanned as part of the Sure Smile procedure. A computerized representation of the patient's teeth is created using the information utilizing an intra-oral scan. To enable greater slot dimension accuracy, a 3D library containing the exact products’ measurements for every bracket is placed over the scanned brackets. The teeth can then be placed in the exact location that is required. The program uses the specific position of a bracket slot on each tooth to determine the archwire bends required to produce the final dental setup when the orthodontist verifies the digital dental setup.

C*ustomized Brackets*

Intraoral scanning of the patient's entire teeth begins off the procedure. A digital buccal-lingual border is created using the intraoral scan's soft tissue contour. A digital setting for the correct arch form and occlusion is then finished by the orthodontic technicians and delivered to the clinician for approval. One of the first products on the market, Insignia software gave the practitioner the ability to modify the virtual setup to fine-tune the 3D positioning of specific teeth, alteration of arch form, change the arc as necessary, and specify all the tooth relations in centric relation. The Insignia system's next procedure is to accurately set the personalized bracket on each tooth in the right location to maximize the efficiency of the unique appliance. To position the appliances indirectly, bracket transfer jigs are specifically cut to match the occlusal areas of the tooth.

Retention and outcome assessment

CAD/CAM Technology for Retainer Manufacturing

**Figure 4:**
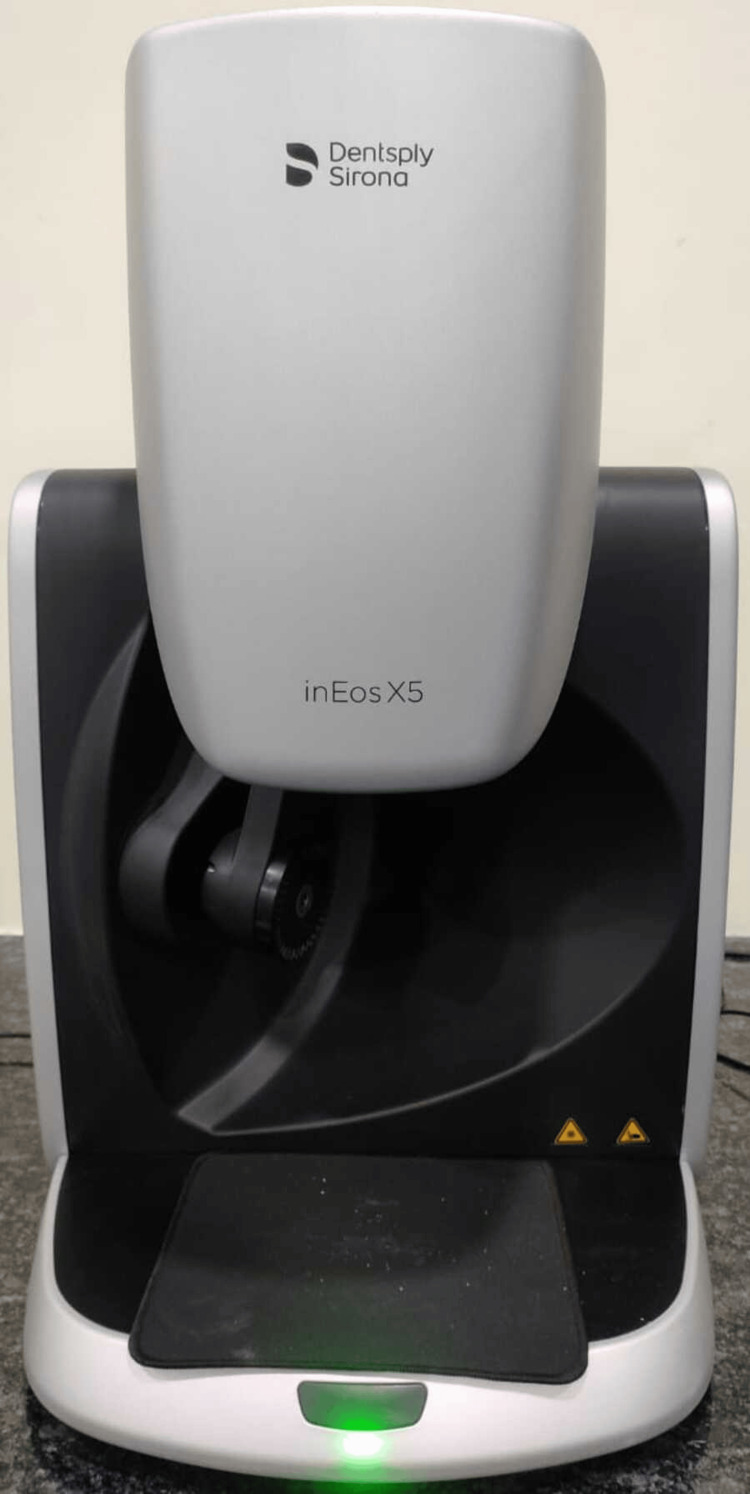
CAD/CAM scanner Picture credit - Samiksha Thawri

In the published research, it has been documented that orthodontic appliances are designed and made using CAD/CAM technology. By using this technology, specialized brackets, Herbst appliances, lingual arches, removable appliances, occlusal splints, and other devices have been created. Various approaches have been reported with comparable first stages. Dental casts are first digitalized, and then the appliances are created using 3D software. The technology used to create the virtual appliance can be additive or subtractive. By machining, the appropriate pattern from a material block an appliance is created using the subtractive approach. Contrarily, the additive process involves layering material to create the desired design all the way through. The additive method of 3D printing is called stereolithography. In terms of quality, expense, speed, and the ability to produce complicated structures, this type of 3D printing is favored for subtractive methods [34]. After the orthodontic treatment is complete, fixed retainers are applied lingually, typically extending from canine to canine. The retainers can be three-dimensionally manufactured, by doing so the placement precision is increased [35]. Figure 4 shows CAD/CAM scanners for the selection process of the article. 

3D Prognosis of Arch After Ortho Treatment

To get stable outcomes from orthodontic therapy, the initial shape of the tooth arch should be considered. The arch form should be assessed during the diagnosis stage, and the archwire utilized should then be selected based on how well it complements each one of these designs. This method is prone to errors because it depends only on the operators' perspective. As a result, 3D software has been created to aid in selecting appropriate archwires for each specific scenario. 3D systems have been shown to improve the evaluation of arch form precision and ultimate prognosis when compared to the traditional method. Additionally, these devices can give the orthodontist quantitative information that compares the dental arch and the arch wires that are currently in use [36].

## Conclusions

The use of 3D technology in the designing and manufacturing orthodontic equipment has significantly advanced the field of orthodontic technology in recent decades. There is a variety of clinical data that supports the efficacy and efficiency of different appliances, and no one system stands out as being much more successful. To further understand the technology and how it should be used, more investigation into the benefits and drawbacks of the existing CAD/CAM is required.

Recent years have seen a rapid advancement in 3D technologies, which has greatly improved orthodontics. These developments lead to an improved workflow for any specific patient's diagnosis, treatment planning, case monitoring, and outcome evaluation. The orthodontist can improve the quality of the treatment they deliver by making the most of their time and knowledge by thoroughly studying these technologies. Patient care will ultimately improve as these 3D systems will continue to develop to meet the requirements of the professionals.

## References

[1] Martin CB, Chalmers EV, McIntyre GT, Cochrane H, Mossey PA (2015). Orthodontic scanners: what's available?. J Orthod.

[2] Impellizzeri A, Horodynski M, De Stefano A (2020). CBCT, and intra-oral scanner: the advantages of 3D technologies in orthodontic treatment. Int J Environ Res Public Health.

[3] Son K, Lee WS, Lee KB (2019). Prediction of the learning curves of 2 dental CAD software programs. J Prosthet Dent.

[4] Solem RC (2017). Utilizing three-dimensional data in orthodontic practice and research. Orthod Craniofac Res.

[5] Manosudprasit A, Haghi A, Allareddy V, Masoud MI (2017). Diagnosis and treatment planning of orthodontic patients with 3-dimensional dentofacial records. Am J Orthod Dentofacial Orthop.

[6] Karatas OH, Toy E (2014). Three-dimensional imaging techniques: a literature review. Eur J Dent.

[7] Al Mortadi N, Eggbeer D, Lewis J, Williams RJ (2012). CAD/CAM/AM applications in the manufacture of dental appliances. Am J Orthod Dentofacial Orthop.

[8] Hartwich RM, Prager T, Brinkmann PJ (2007). Suresmile-CAD/CAM system for orthodontic treatment planning, simulation and fabrication of customized archwires. Int J Comp Dent.

[9] Mayhew MJ (2005). Computer-aided bracket placement for indirect bonding. J Clin Orthod.

[10] Gracco A, Tracey S (2011). The insignia system of customized orthodontics. J Clin Orthod.

[11] (2021). Radiation protection no 172: cone beam CT for dental and maxillofacial radiology. Evidence based guidelines. http://www.sedentexct.eu/files/radiation_protection_172.pdf.

[12] Juerchott A, Freudlsperger C, Weber D (2020). In vivo comparison of MRI- and CBCT-based 3D cephalometric analysis: beginning of a non-ionizing diagnostic era in craniomaxillofacial imaging?. Eur Radiol.

[13] Pinheiro M, Ma X, Fagan MJ, McIntyre GT, Lin P, Sivamurthy G, Mossey PA (2019). A 3D cephalometric protocol for the accurate quantification of the craniofacial symmetry and facial growth. J Biol Eng.

[14] Pittayapat P, Limchaichana-Bolstad N, Willems G, Jacobs R (2014). Three-dimensional cephalometric analysis in orthodontics: a systematic review. Orthod Craniofac Res.

[15] Cossellu G, Biagi R, Sarcina M, Mortellaro C, Farronato G (2015). Three-dimensional evaluation of upper airway in patients with obstructive sleep apnea syndrome during oral appliance therapy. J Craniofac Surg.

[16] Schendel SA, Broujerdi JA, Jacobson RL (2014). Three-dimensional upper-airway changes with maxillomandibular advancement for obstructive sleep apnea treatment. Am J Orthod Dentofacial Orthop.

[17] Brunetto DP, Velasco L, Koerich L, Araújo MT (2014). Prediction of 3-dimensional pharyngeal airway changes after orthognathic surgery: a preliminary study. Am J Orthod Dentofacial Orthop.

[18] Görgülü S, Gokce SM, Olmez H, Sagdic D, Ors F (2011). Nasal cavity volume changes after rapid maxillary expansion in adolescents evaluated with 3-dimensional simulation and modeling programs. Am J Orthod Dentofacial Orthop.

[19] Francisco I, Ribeiro MP, Marques F (2022). Application of three-dimensional digital technology in orthodontics: the state of the art. Biomimetics (Basel).

[20] Rheude B, Lionel Sadowsky P, Ferriera A, Jacobson A (2005). An evaluation of the use of digital study models in orthodontic diagnosis and treatment planning. Angle Orthod.

[21] Rossini G, Parrini S, Castroflorio T, Deregibus A, Debernardi CL (2016). Diagnostic accuracy and measurement sensitivity of digital models for orthodontic purposes: a systematic review. Am J Orthod Dentofacial Orthop.

[22] El-Timamy AM, El-Sharaby FA, Eid FH, Mostafa YA (2016). Three-dimensional imaging for indirect-direct bonding. Am J Orthod Dentofacial Orthop.

[23] Möhlhenrich SC, Brandt M, Kniha K (2020). Suitability of virtual plaster models superimposed with the lateral cephalogram for guided paramedian orthodontic mini-implant placement with regard to the bone support. J Orofac Orthop.

[24] Cassetta M, Giansanti M (2016). Accelerating orthodontic tooth movement: a new, minimally-invasive corticotomy technique using a 3D-printed surgical template. Med Oral Patol Oral Cir Bucal.

[25] Maino BG, Paoletto E, Lombardo L 3rd, Siciliani G (2016). A three-dimensional digital insertion guide for palatal miniscrew placement. J Clin Orthod.

[26] Lee RJ, Weissheimer A, Pham J (2015). Three-dimensional monitoring of root movement during orthodontic treatment. Am J Orthod Dentofacial Orthop.

[27] Donaldson CD, Manisali M, Naini FB (2021). Three-dimensional virtual surgical planning (3D-VSP) in orthognathic surgery: advantages, disadvantages and pitfalls. J Orthod.

[28] Aristizábal JF, Martínez-Smit R, Díaz C, Pereira Filho VA (2018). Surgery-first approach with 3D customized passive self-ligating brackets and 3D surgical planning: case report. Dental Press J Orthod.

[29] Vale FD, Scherzberg J, Cavaleiro J, Sanz D, Caramelo F, Malo L, Marcelino JP (2016). 3D virtual planning in orthognathic surgery and CAD/CAM surgical splints generation in one patient with craniofacial microsomia: a case report. Dent Press J Orthod.

[30] Elnagar MH, Aronovich S, Kusnoto B (2020). Digital workflow for combined orthodontics and orthognathic surgery. Oral Maxillofac Surg Clin North Am.

[31] Otranto de Britto Teixeira A, Almeida MA, Almeida RC (2020). Three-dimensional accuracy of virtual planning in orthognathic surgery. Am J Orthod Dentofacial Orthop.

[32] Tonin RH, Iwaki Filho L, Yamashita AL (2020). Accuracy of 3D virtual surgical planning for maxillary positioning and orientation in orthognathic surgery. Orthod Craniofac Res.

[33] Larson BE, Vaubel CJ, Grünheid T (2013). Effectiveness of computer-assisted orthodontic treatment technology to achieve predicted outcomes. Angle Orthod.

[34] Nasef AA, El-Beialy AR, Mostafa YA (2014). Virtual techniques for designing and fabricating a retainer. Am J Orthod Dentofacial Orthop.

[35] Wolf M, Schumacher P, Jager F (2015). Novel lingual retainer created using CAD CAM technology Evaluation of its positioning accuracy Neuer CAD CAM gefertigter Lingualretainer. J Orofac Orthop.

[36] Asquith JA, McIntyre GT (2012). Dental arch relationships on three-dimensional digital study models and conventional plaster study models for patients with unilateral cleft lip and palate. Cleft Palate Craniofac J.

